# Spikes on ripples are better interictal biomarkers of epilepsy than spikes or ripples

**DOI:** 10.1093/braincomms/fcaf056

**Published:** 2025-02-08

**Authors:** Lorenzo Fabbri, Margherita A G Matarrese, Saeed Jahromi, Michael Scott Perry, Eleonora Tamilia, Joseph R Madsen, Scellig S D Stone, Phillip L Pearl, Christos Papadelis

**Affiliations:** Neuroscience Research, Jane and John Justin Institute for Mind Health, Cook Children’s Health Care System, Fort Worth, TX 76104, USA; Department of Bioengineering, The University of Texas at Arlington, Arlington, TX 76010, USA; Neuroscience Research, Jane and John Justin Institute for Mind Health, Cook Children’s Health Care System, Fort Worth, TX 76104, USA; Department of Bioengineering, The University of Texas at Arlington, Arlington, TX 76010, USA; Research Unit of Intelligent Health Technology for Health and Wellbeing, Department of Engineering, Università Campus Bio-Medico di Roma, Rome 00128, Italy; Neuroscience Research, Jane and John Justin Institute for Mind Health, Cook Children’s Health Care System, Fort Worth, TX 76104, USA; Department of Bioengineering, The University of Texas at Arlington, Arlington, TX 76010, USA; Neuroscience Research, Jane and John Justin Institute for Mind Health, Cook Children’s Health Care System, Fort Worth, TX 76104, USA; Fetal-Neonatal Neuroimaging and Developmental Science Center, Boston Children’s Hospital, Harvard Medical School, Boston, MA 02115, USA; Division of Epilepsy and Clinical Neurophysiology, Department of Neurology, Boston Children’s Hospital, Harvard Medical School, Boston, MA 02115, USA; Division of Epilepsy Surgery, Department of Neurosurgery, Boston Children’s Hospital, Harvard Medical School, Boston, MA 02115, USA; Division of Epilepsy Surgery, Department of Neurosurgery, Boston Children’s Hospital, Harvard Medical School, Boston, MA 02115, USA; Division of Epilepsy and Clinical Neurophysiology, Department of Neurology, Boston Children’s Hospital, Harvard Medical School, Boston, MA 02115, USA; Neuroscience Research, Jane and John Justin Institute for Mind Health, Cook Children’s Health Care System, Fort Worth, TX 76104, USA; Department of Bioengineering, The University of Texas at Arlington, Arlington, TX 76010, USA; School of Medicine, Texas Christian University, Fort Worth, TX 76109, USA

**Keywords:** spikes, high-frequency oscillations, epilepsy biomarkers, epilepsy surgery

## Abstract

Spikes are the most established interictal epilepsy biomarkers. Yet, they suffer from low specificity since they are partially concordant with the epileptogenic zone and are often found in non-epileptogenic areas. High-frequency oscillations, classified as ripples and fast ripples, are considered more specific biomarkers compared with spikes. Ripples occur more often than fast ripples but are believed to be less specific, since they are more frequently generated by physiological mechanisms. Here, we examine the temporal relationship between spikes, ripples and fast ripples, and assess the ability of these biomarkers (and their combinations) to delineate the epileptogenic zone and predict outcome. We hypothesize that spikes on ripples (temporal co-occurrence of spikes and ripples) can identify the epileptogenic zone and predict outcome better than spikes or ripples.

We analysed intracranial EEG data from 40 children with drug-resistant epilepsy. Spikes, ripples and fast ripples were classified based on their temporal occurrence. Their rates were compared with resection by performing a receiver operating characteristic analysis. The resection ratio, quantifying the extent of each biomarker’s removal, was computed, and correlated with patients’ outcome.

Spikes on ripples were seen in all patients; fast ripples were seen in 43% of patients. In good outcome patients, fast ripple and spike on ripple rates were higher inside resection (*P* = 0.027; *P* = 0.003, respectively). Fast ripples and spikes on ripples resection ratio predicted outcome (*P* < 0.05). For fast ripples, outcome was predicted in 82% of patients; this proportion was higher than the one for spikes (48%, *P* = 0.015) and ripples (40%, *P* = 0.003), and spikes on ripples (53%, *P* = 0.034). Fast ripples were the most accurate (82%) to predict outcome; spikes on ripples were the most precise (positive predictive value = 90%). Spike rate and spikes on ripples performance to predict the epileptogenic zone were correlated (*r* = 0.36, *P* = 0.035). For patients with frequent spikes, spikes on ripples accuracy to predict outcome reached 70%.

Fast ripples are the best biomarker, but they can be seen in only half of patients with drug-resistant epilepsy. Spikes on ripples are a good alternative with more universal applicability since they can be seen in all patients while their resection predicts good outcome; their performance is improved in patients with frequent spikes. Overall, in the absence of fast ripples, spike on ripple areas should be targeted during surgery.

## Teaser

Fabbri *et al*. report that spikes on ripples are promising biomarkers for identifying the epileptogenic zone and predicting outcome in drug-resistant epilepsy, especially when fast ripples are not present. They show potential for universal applicability and improved performance in patients with frequent spikes.

## Graphical Abstract

**graphical abstract:**
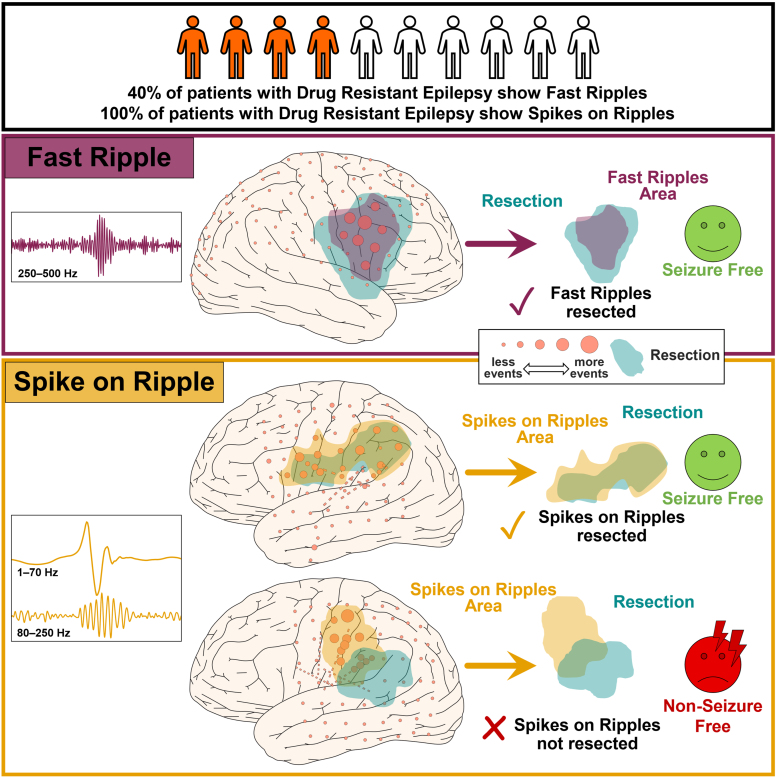

## Introduction

Epilepsy surgery is the most effective treatment for patients with focal drug-resistant epilepsy (DRE).^1,2^ Its success relies on the complete resection (or disconnection/ablation) of the epileptogenic zone (EZ), the brain area that is essential for generating seizures. Currently, the best approximator of the EZ is the seizure onset zone (SOZ), the brain area where most of the seizures are initiated, determined through invasive electroencephalographic (iEEG) recordings. Yet, seizures are unpredictable and may take several hours or days to occur at the expense of considerable human and financial resources.^3^ Thus, there is an urgent need for an interictal biomarker that delineates the EZ from iEEG recordings without having to wait for a seizure to occur.^4^

Spikes are the most established interictal biomarkers of epilepsy.^5^ Yet, they suffer from low specificity since they are partially concordant with the EZ and are often found in non-epileptogenic areas that should not be resected during surgery.^6^ High-frequency oscillations (HFOs)^7^ are considered more specific biomarkers of the EZ compared with spikes.^8^ Based on their frequency, they are classified into ripples (80–250 Hz) and fast ripples (250–500 Hz).^9,10^ Fast ripples are often associated with the EZ but are less frequently observed in patients with DRE^11-13^ while their low sensitivity may hinder the EZ localization.^14,15^ In contrast to fast ripples, ripples are present in most patients with DRE^16-18^ with higher rates than fast ripples.^19^ However, not all ripples are pathological as they can also occur in physiological brain areas, such as the hippocampus, the occipital cortex and paracentral areas.^20-26^ Several iEEG studies have shown that the presence of residual spikes,^27^ ripples^8^ and fast ripples^28-30^ following surgery is linked to poor outcome. Contrarily, resection of cortical areas generating fast ripples^31-34^ or ripples^8,31,35^ is associated with good outcome. At the group level, localization of HFOs correlates better with the SOZ and surgical outcome than localization of spikes.^28,32,36,37^ Yet, at the patient’s level, removal of fast ripples areas does not always predict outcome, while in some patients, who are free of seizures, areas with high rates of fast ripples remain intact.^38-40^ Moreover, a recent randomized, single-blind, clinical trial showed that HFOs are not superior to spikes on intraoperative electrocorticography (ECoG) for tailoring epilepsy surgery, both overall and by epilepsy type (i.e. temporal and extratemporal lobe epilepsy).^41^ This trial challenged the clinical value of HFOs as an interictal biomarker of epilepsy. In summary, existing evidence indicates that there is currently no single interictal biomarker that allows the unambiguous definition of the EZ.

Spikes and HFOs seem to represent independent neurophysiological events. Yet, they often co-occur in time.^42^ A conspicuous body of literature showed that spikes temporally co-occurring with ripples (S + R) are superior epilepsy biomarkers compared with spikes only.^43-46^ Good outcome patients had a greater proportion of S + R removed compared with poor outcome,^43,44^ and the proportion of S + R removed was higher than other biomarkers.^43^ Contrarily, other studies found no statistical support for HFOs (or their variants) over spikes in defining the EZ,^14^ and reported that HFOs were not more accurate than spikes in predicting outcome.^47^ Yet, these studies presented with several limitations. Roehri *et al*. did not consider surgical outcome as the gold standard for defining the EZ, while Gerstl *et al*. defined biomarker areas using a statistical threshold. This approach can be useful to reduce the number of contacts in the area to investigate but may penalize patients with an extensive EZ as it defines restricted areas with elevated biomarker rates; it also requires validation and optimization in larger studies encompassing diverse pathologies. Thus, there is a need to further investigate combinations of interictal biomarkers for delineating the EZ and predict outcome.

The goal of this study is to examine the temporal relationship between spikes, ripples and fast ripples, and assess the ability of these biomarkers (and their combinations) to delineate the area to resect and predict surgical outcome in children with DRE. We hypothesize that S + R (i.e. temporal co-occurrence of spikes and ripples) can identify the EZ and predict surgical outcome better than spikes or ripples. To test our hypothesis, we automatically detected spikes, ripples and fast ripples on iEEG data recorded from children with DRE, classified them as co-occurring or in isolation based on their latency, compared their performance in predicting the SOZ and resected area (RA) in good outcome patients (at the group and patient’s level), and correlated their removal with patient’s outcome.

## Materials and methods

### Patients

We retrospectively reviewed patients with DRE who underwent resective surgery at Boston Children’s Hospital (BCH) between February 2011 and June 2018. We selected patients satisfying the following criteria: (i) long-term iEEG monitoring with grids, strips, and/or depth electrodes; (ii) iEEG sampling rate ≥ 1000 Hz;^48^ (iii) availability of surgical outcome at least 1 year after surgery; and (iv) availability of pre- and post-surgical MRI and post-implantation computerized tomography (CT). The study was approved by the North Texas Regional Institutional Review Board (2019–166; Principal Investigator: Christos Papadelis).

### Structural imaging and intracranial EEG recordings

We utilized MRI and CT scans to gather structural information for each patient. Using *Brainstorm*,^49^ the pre-implant MRI space was used as the reference space to rotate and reslice the post-implant CT (voxel size = 0.5 × 0.5 × 0.5 mm^3^) and post-surgical MRI. Additionally, we segmented the preoperative MRI and reconstructed the patient-specific 3D cortical surface through *FreeSurfer*.^50^ Electrode implantation was planned to monitor all ‘areas of interest’ regarded as possibly epileptogenic based on patient’s presurgical evaluation. Intracranial EEG was obtained with subdural grids (2.3 mm diameter and 10 mm inter-contact distance) and/or depth electrodes (6–16 linearly arranged contacts with ∼1 mm diameter and ∼3–5 mm inter-contact distance) (Ad-Tech., USA) using a Natus Quantum acquisition system (Natus Inc., USA) with an antialiasing filter set to half of the sampling frequency. Per BCH’s clinical practice, antiseizure medications were withdrawn or adjusted during the iEEG monitoring to maximize the likelihood of capturing relevant seizure activity. A list of antiseizure medications is shown in Supplementary Table 1. Independently from this study, we defined the position of the implanted electrodes through a three-step procedure described previously.^51^ We identified epochs of non-REM slow-wave sleep (when this was possible) with minimal presence of motion artefacts. The epochs were selected to be at least 1 h before/after a clinical seizure or half an hour before/after an electrographic seizure.^52^

### SOZ, RA and surgical outcome

Pediatric epileptologists defined the SOZ independently from this study during each patient’s long-term iEEG monitoring. The iEEG contact(s) showing the earliest change in activity associated with clinical or subclinical seizures were identified as the SOZ. The RA was acquired by delineating the post-surgical cavity boundaries on the preoperative MRIs.^49^ The resected contact(s) were defined as the contacts located inside or on the edges of the RA. Patient outcome was assessed based on the patient’s most recent follow-up at least 1 year after surgery using the Engel classification (Classes I–IV).^53^ We then categorized patients into having good (Engel IA-D) or poor (Engel ≥ II) outcome.

### Automated detection of spikes and HFOs

Spikes were detected on each channel using Persyst 14.0 (Persyst Development).^54^ Detection was performed for each channel separately on preprocessed segments (common average reference, DC removal, band-pass between 1–70 and 60 Hz notch). Prior results indicated that Persyst demonstrated statistical non-inferiority to human readers in terms of sensitivity and false positive (FP) rate.^54^ We validated Persyst spike detection with respect to the markings of an EEG expert (C.P.) using a previously described procedure^55^ on 5 min data from five patients and obtained a sensitivity of 85% and precision of 72%. HFOs were automatically detected on each channel using the *RippleLab* Hilbert detection algorithm^56^ implemented in MATLAB (The MathWorks, Inc.). Detection of ripples and fast ripples was performed separately; the parameters used for detection are reported in Supplementary Table 2. To further reduce false positive detections, we performed a visual inspection by an experienced reviewer (C.P.) following the latest recommendations for HFOs detection.^19,57-59^ We considered ‘true HFOs’ events showing either an isolated peak in the time-frequency plane or at least four oscillations in the filtered signal. Events were rejected if showing an ‘elongated blob’ in frequency in the time-frequency plane and no oscillations in the filtered signal (typically associated with artefacts) or if they were related to sharp transients after filtering caused by the Gibbs phenomenon.^60^

### Temporal categorization of spikes and HFOs

**Figure 1:**
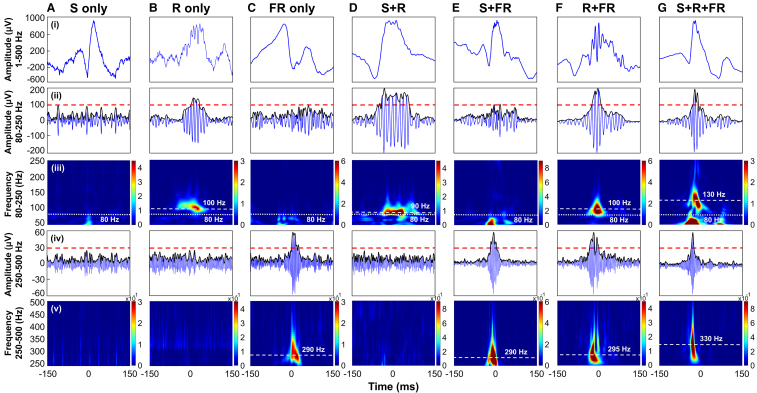
**Categories of events based on time-frequency analysis classification.** Example of biomarkers on iEEG recordings (300 ms) for 7 out of 11 categories of events (S only, R only, FR only, S + R, S + FR, R + FR and S + R + FR). Each scenario shows a biomarker in (**I**) unfiltered iEEG (1st row); (ii) band-pass data in the frequency band 80–250 Hz (2nd row); (iii) time-frequency domain where ripples are seen as an island in the spectral content within the ripple frequency band (80–250 Hz) (3rd row); (iv) band-pass data in the frequency band 250–500 Hz (4th row) and (**V**) t**I**me-frequency domain where fast ripples are seen as an island in the spectral content within the fast ripple frequency band (250–500 Hz) (5th row). The detected events were visually inspected by two independent reviewers to exclude artefacts. In the filtered time domain (2nd and 4th rows), the black line represents the envelope of the analytic signal obtained using the Hilbert transform. The red dashed line represents the threshold value above which an event is considered a valid HFO. The white line in the time-frequency domain identifies the peak frequency for the HFO. S = spike; R = ripple; FR = fast ripple.

By using an in-house custom algorithm, spikes and HFOs (either ripples or fast ripples) were divided into 11 categories: all spikes (All S), all ripples (All R), all fast ripples (All FR), spike only (S only), ripple only (R only), fast ripple only (FR only), spike + ripple (S + R), spike + fast ripple (S + FR), ripple + fast ripple (R + FR), spike + ripple + fast ripple (S + R + FR) and spike + HFO (S + HFO). S + HFO events were defined as spikes co-occurred with either a ripple or fast ripple on the same channel only. Spikes and HFOs were detected separately and classified into their subclasses using an in-house algorithm that looked at the time stamps of each event. Events were considered as co-occurring if appearing within a ± 50 ms window; otherwise, they were regarded as isolated events. Figure 1 displays examples of S, R, FR only, S + R, S + FR, R + FR and S + R + FR along with their corresponding time-frequency analysis plots. We estimated the occurrence rate of these biomarkers by dividing the count of these events by the length of each patient’s iEEG recordings and used this rate in further analyses. We defined the area percentage as the proportion of resected, SOZ, and channels showing a specific biomarker over the total number of implanted channels. We compared the rates of All S, All R and All FR across the following main lobes: temporal, occipital, parietal and frontal. We categorized each contact into a specific lobe based on the iEEG implantation plan. In cases where an electrode spanned multiple lobes, we relied on co-registered CT images to establish the specific lobe where each contact was situated. In addition, we investigated whether different groups of underlying conditions influenced biomarker rates by comparing All S, All R and All FR rates across three categories: developmental, acquired and non-lesional, as defined in Table 1.

**Table 1:** Patients’ demographics and clinical characteristics

Patient	Sex	Age [yr.]	IcEEG Type, # (SE + DE)	Side [L/R]	MRI Findings	Pathology	Surgical outcome (Engel)	P/o follow-up [yrs.]	% Brain volume resected
**1**	M	10	SE (80)	R	Normal	NL	Good (1a)	5	1.41
**2**	M	17	SE (140)	L/R	Normal	NL	Good (1a)	2	4.18
**3**	M	10	98 (88 + 10)	L	Normal	NL	Good (1a)	4	0.95
**4**	F	7	DE (90)	L	FCD (T and Ins)	DEV	Good (1b)	8	1.61
**5**	F	13	SE (72)	L	Normal	NL	Good (1a)	5	1.82
**6**	M	17	92 (72 + 20)	L	Tumour (T)	ACQ	Good (1c)	6	3.28
**7**	F	16	106 (96 + 10)	L	FCD (T)	DEV	Good (1a)	8	2.27
**8**	M	2	SE (112)	R	TSC (multifocal)	DEV	Good (1a)	7	7.42
**9**	M	2.25	SE (80)	L	Frontal lesion	ACQ	Good (1a)	6	2.59
**10**	M	19	SE (64)	L	FCD (mesial T)	DEV	Good (1a)	4	2.67
**11**	F	18	SE (88)	R	Normal	NL	Good (1a)	7	2.64
**12**	M	15	SE (80)	L	Hippocampal Sclerosis (anterior T	DEV	Good (1a)	2	2.19
**13**	M	15	SE (88)	L	Normal	NL	Good (1a)	5	1.11
**14**	F	18	154 (144 + 10)	L	FCD (F)	DEV	Good (1a)	3	0.68
**15**	M	3	SE (96)	L	FCD (T)	DEV	Good (1a)	1.5	7.92
**16**	M	13	102 (72 + 30)	L	Encephalomalacia (*P*, superior T)	ACQ	Good (1c)	2	1.26
**17**	F	3	SE (120)	L/R	TSC (multifocal)	DEV	Good (1a)	2	2.81
**18**	M	4	66 (56 + 10)	L/R	FCD (F)	DEV	Good (1c)	6	2.86
**19**	M	13	SE (136)	L	Infarct (MCA territory)	ACQ	Good (1c)	5	5.38
**20**	M	22	94 (64 + 30)	L	FCD (C, *P*)	DEV	Good (1a)	3	2.12
**21**	F	18	112 (72 + 40)	L	FCD (F)	DEV	Good (1a)	2	0.55
**22**	F	7	100 (80 + 20)	L	Parietal lesion	ACQ	Good (1a)	1	0.69
**23**	F	12	DE (196)	L/R	PMG (T,P,O)	DEV	Good (1a)	1	5.83
**24**	M	15	DE (236)	L	Normal	NL	Good (1c)	5	3.07
**25**	F	4	96 (72 + 24)	L	Inferior Fr sulcus, pars Tr	DEV	Good (1a)	5	1.95
**26**	F	4	DE (162)	R	Frontal lesion	ACQ	Good (1a)	1	0.68
**27**	F	9	DE (140)	L	Hippocampal sclerosis (mesial T, periventricular)	DEV	Poor (4b)	6	0.96
**28**	F	9	100 (80 + 20)	R	FCD (P)	DEV	Poor (3a)	8.5	2.31
**29**	M	6	SE (120)	L	FCD (F)	DEV	Poor (3a)	11	2.61
**30**	F	5	168 (128 + 40)	L/R	TSC (P,O)	DEV	Poor (3a)	2	17.1
**31**	F	13	122 (112 + 10)	L/R	Normal	NL	Poor (3)	1	1.19
**32**	M	17	SE (128)	R	Glioma (PO junction)	ACQ	Poor (3a)	4	1.45
**33**	F	15	SE (72)	L	FCD (mesial P)	DEV	Poor (3a)	6	0.86
**34**	M	12	122 (112 + 10)	L	FCD (T)	DEV	Poor (3a)	6	7.75
**35**	M	16	DE (212)	L	FCD (superior F gyr.)	DEV	Poor (3a)	6	0.40
**36**	F	22	SE (120)	L/R	Normal	NL	Poor (2b)	5	2.85
**37**	M	10	124 (64 + 60)	L	PMG (F,P)	DEV	Poor (3a)	7	6.16
**38**	F	6	SE (120)	R	FCD	DEV	Poor (2b)	8	0.94
**39**	M	4	138 (128 + 10)	L/R	FCD (F)	DEV	Poor (3a)	4	7.94
**40**	M	15	DE (162)	L	FCD (T,O)	DEV	Poor (3)	1	4.38

F, Female; M, Male; Age, age at epilepsy surgery; DE, depth electrodes (stereo EEG); SE, surface electrodes (electrocorticography); L, Left; R, Right; T, Temporal; F, Frontal; *P*, Parietal; T, Temporal; O, Occipital; Ins, Insula; Tr, triangularis; FCD, focal cortical dysplasia; TSC, tuberous sclerosis complex; MCA, middle cerebral artery; PMG, polymicrogyria; NL, Non-lesional; DEV, Malformation of Cortical Development (i.e. focal cortical dysplasia, polymicrogyria, tuberous sclerosis complex, dysembryoplastic neuroepithelial tumour, and glioma); ACQ, Acquired (i.e. stroke, neoplasm, and traumatic brain injury); yrs, years.

### SOZ and RA prediction

For each biomarker rate, we performed the receiver operating characteristic (ROC) curve across different thresholds to evaluate its performance of localizing the RA and SOZ in good outcome patients (Engel I) whose resected regions were confirmed to be epileptogenic. The RA was defined as the resected/ablated contacts in good outcome patients only. We defined as true positives (TPs), contacts that were within RA (or SOZ) showing rates greater than a set threshold; as true negative (TN), contacts that were outside the RA (or SOZ) showing rates lower than a set threshold; as FPs, contacts that were within the RA (or SOZ) showing rates lower than a set threshold, and as false negatives (FNs), contacts that were outside the RA (or SOZ) showing rates higher than a set threshold. To minimize the number of FPs contacts, we further evaluated sensitivity values by setting the specificity in the range of 85–95% (step 5%). Moreover, we evaluated each biomarker’s ability to accurately delineate the RA (or the SOZ) by studying its performance individually for each patient. Particularly, the partial area under the curve (pAUC) was calculated for the specificity range of 85−100% and then normalized by dividing by the maximum possible area (0.15), resulting in an index that ranges from 0 to 1. The chance level for the pAUC is 0.5 corresponding to the maximum possible pAUC value divided by 2 (i.e. 0.075).

### Prediction of surgical outcome

We computed the resection ratio^44^ for each biomarker and the SOZ ratio^44^ defined as the following equations:


(1) $$\mathrm{Resection}\;\mathrm{Ratio}\;(\mathrm{BM})=\left(\frac{\sum_{\mathrm{res}}\mathrm{Rat}e_{\mathrm{BM}}}{\sum_{[\mathrm{res},\mathrm{non}\text{-}\mathrm{res}]}\mathrm{Rat}e_{\mathrm{BM}}}\right)$$

(2) $$\mathrm{SOZ}\;\mathrm{Ratio}=\left(\frac{\#\;\;\mathrm{ChannelsSO}Z_{\mathrm{res}}}{\#\;\;\mathrm{ChannelsSO}Z_{\mathrm{res}}+\;\#\;\;\mathrm{ChannelsSO}Z_{\mathrm{non}\text{-}\mathrm{res}}}\right)$$

where BM indicates the type of biomarker and *res* and *non-res* correspond to iEEG contacts classified as either ‘resected’ or ‘non-resected’, respectively. For the resection ratio, the numerator in Eq. (1) represents the summation of the rates of resected iEEG contacts, whereas the denominator represents the summation of the rates of both resected and non-resected iEEG contacts. For the SOZ ratio, the numerator in Eq. (2) represents the number of resected iEEG contacts within the SOZ, while the denominator represents the number of both resected and non-resected iEEG contacts within the SOZ. A resection ratio value close to 1 indicates that all events of a specific biomarker are removed, whereas a resection ratio value close to 0 indicates that none of its events are removed. A SOZ ratio value close to 1 indicates that all the SOZ was removed, whereas a SOZ ratio value close to 0 indicates that none of the SOZ was removed. To test whether complete resection of a specific biomarker could predict outcome, we performed a ROC analysis and computed for each biomarker the sensitivity (TP/[TP + FN]), specificity (TN/[TN + FP]), positive (TP/[TP + FP]) and negative predictive values (TN/[TN + FN]), markedness (PPV + NPV-1), false positive rate (FP/[FP + TN]) and accuracy ([TP + TN]/[TP + FP + TN + FN]) obtained at the optimal receiver operating point (highest Youden’s Index). Additionally, we conducted a further analysis of outcome prediction focusing on two subsets of patients: (i) those with a median spike rate above 0.7 spikes/minute (corresponding to the median spike rate across the whole cohort) and (ii) those with no fast ripples and rare spikes (i.e. spike rate <0.7 spikes/minute). Moreover, we conducted a cross-validated pseudo-prospective prediction of outcome using a resection ratio cut-off threshold of 0.5 to assess the effects of a partial resection on patient’s outcome.

### Statistical analysis

Statistical analysis was performed in MATLAB R2023a (The MathWorks, Inc.). We considered a statistical significance if *P* < 0.05, and reported measures as median (IQR), except otherwise stated. We applied a *Wilcoxon rank sum* test for comparisons of biomarker rates between good (*n* = 26) and poor (*n* = 14) outcome patients. We applied a *Wilcoxon sign-rank* test for paired comparisons of biomarkers rates between inside and outside SOZ/RA, regardless of whether all channels recorded such patterns. The pAUC values and area percentages were compared using a *Kruskal–Wallis* test. *Tukey–Kramer* correction was used for multiple comparisons. We computed the *Spearman* correlation coefficient to assess if a higher spike rate was associated with better performance in predicting the SOZ or RA in terms of pAUC. *Bernard* test was used to evaluate whether a biomarker removal was associated with outcome. Proportions of patients with a specific biomarker or with correctly predicted outcomes were compared using a *two-proportions* z-test. Finally, to reduce the variability associated with our cohort, the ROC analysis for outcome prediction was cross-validated using a leave-one-out cross-fold validation, excluding one patient for each iteration.

## Results

### Cohort

The patient’s clinical findings and demographics are reported in Table 1. We included 40 patients (18 females) with DRE (median age at surgery = 12.5 (6–16) years; Supplementary Table 3). Twenty-six patients (65%) were seizure-free at least 2 years after surgery and were regarded as having a good outcome. The median follow-up was 5 (2–6) years (Supplementary Table 3). The median number of resected and SOZ electrodes were 14.5 (9–26.5) contacts and 9 (4.8–18.5) contacts, respectively (Supplementary Table 3). We analysed a median of 5.4 (5–27.7) minutes of data and 112 (91.5–136.5) contacts per patient. Subdural electrodes were implanted in 17 patients, depth electrodes in seven patients, and both types in 16 patients (33/40 patients had a sampling rate ≥2 kHz). We found no differences in any of the previously described features between good and poor outcome patients.

### Distribution of spikes, ripples and fast ripples

**Figure 2:**
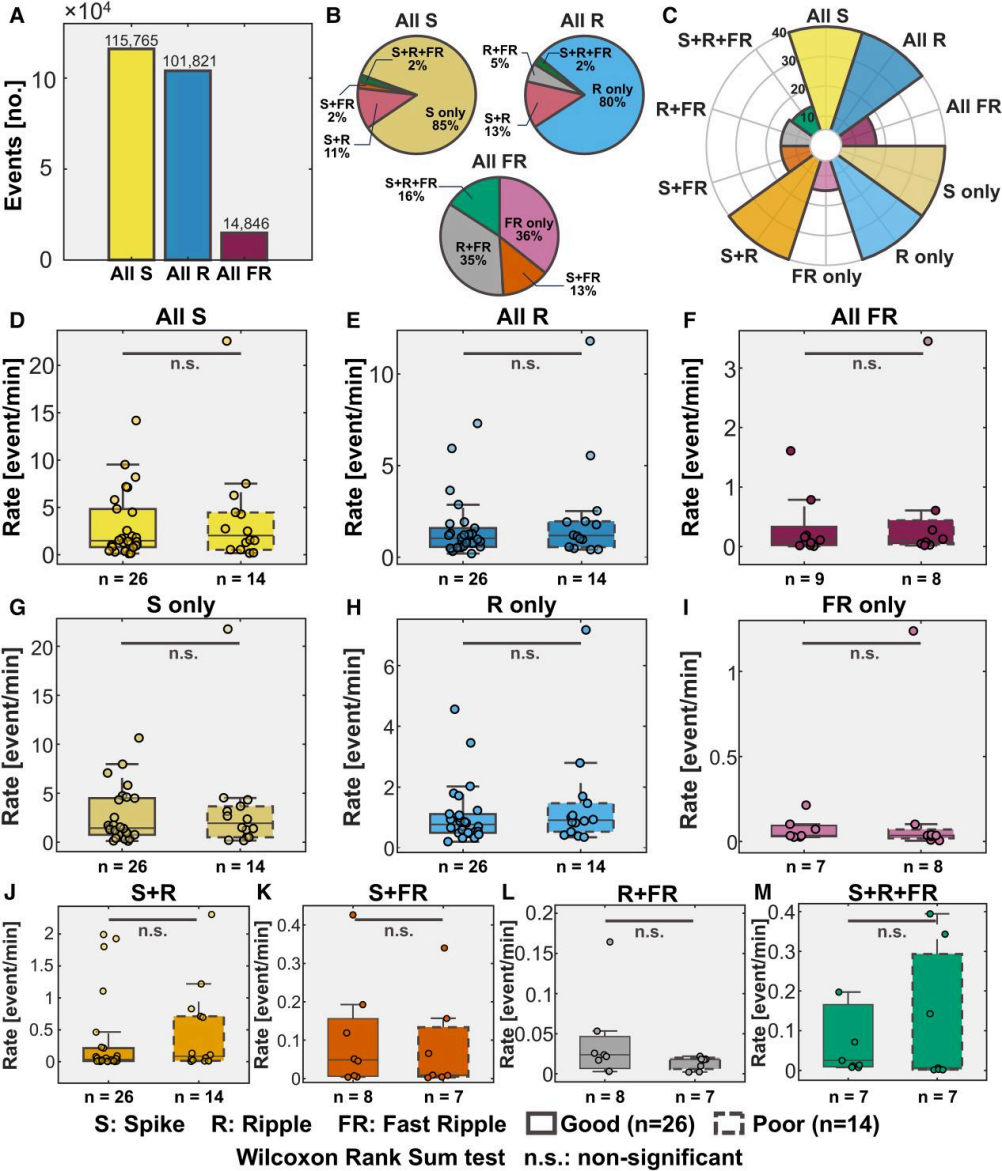
**Cohort characteristics.** Panel **A** represents the total number of spikes, ripples and fast ripples detected in our cohort. Panel **B** shows spikes, ripples and fast ripples broken down in their subgroups and shown as percentages. Panel **C** shows for how many patients a particular biomarker was found. Panels **D** through M show the comparison of biomarker rates between good and poor outcome patients.

We detected a total of 115 765 spikes, 101 821 ripples and 14 846 fast ripples (Fig. 2A). For All S, All R and All FR, 85%, 80% and 36% of them occurred alone, respectively (Fig. 2B). For All S, 11% and 2% of them occurred on a ripple and fast ripple, respectively (Fig. 2B). For All R, 13% and 5% of them occurred on a spike and fast ripple, respectively (Fig. 2B). For All FR, 13% and 35% of them occurred on a spike and ripple, respectively (Fig. 2B). All S, S only, All R, R only and S + R were seen in all patients (Fig. 2C). On the other hand, All FR were seen in 17/40 patients (Fig. 2C, 43%; nine good outcome). S + FR and R + FR were seen in 15/40 patients (Fig. 2C, 38%; eight good outcome). Finally, S + R + FR were seen in 14/40 patients (Fig. 2C, 35%; seven good outcome). The proportion of patients with All FR, S + FR, S + R + FR was not different between good and poor outcomes (33% versus 62%, two-tailed *P* = 0.09, 30% versus 54%, two-tailed *P* = 0.14, 26% versus 54%, two-tailed *P* = 0.08, respectively) while the proportion of patient with FR only and R + FR was different between the two groups (26% versus 62%, two-tailed *P* = 0.03, 26% versus 62%, two-tailed *P* = 0.03, respectively). We found no difference between good and poor outcome patients in the mean rate of all biomarkers (Fig. 2D/M). Moreover, we found no differences in the rates of All S, All R and All FR across different lobes (Supplementary Fig. 1A-C) and across different groups of underlying conditions (Supplementary Fig. 1D-F).

### Biomarkers distribution over resected versus non-RAs

**Figure 3:**
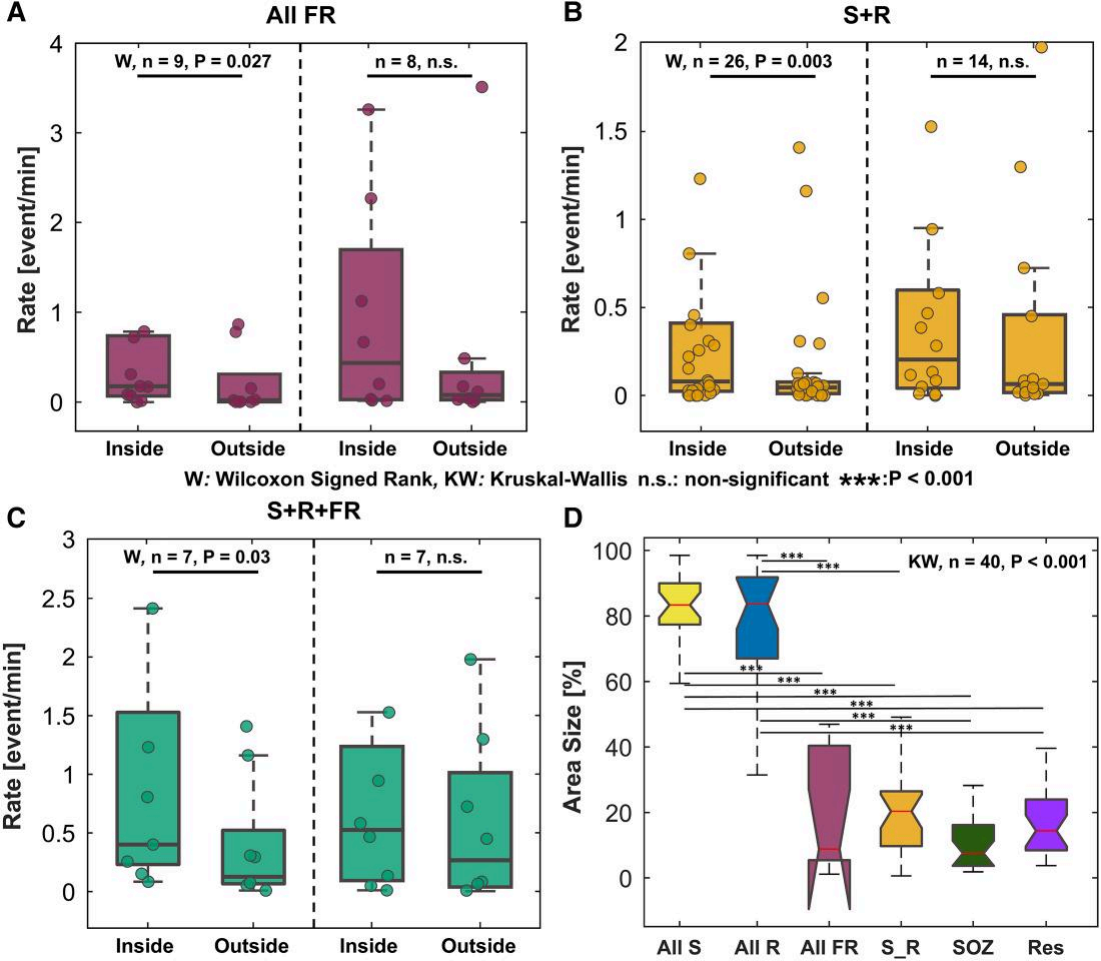
**Rates inside and outside resection and area size results. (A–C)** Rates inside and outside resection for patients with good (left) and poor (right) outcomes. **(D)** Comparison of Area Size between All S, All R, All FR, S + R, SOZ and resection. All S = all spikes; All R = all ripples; All FR = all fast ripples; S + R = spike + ripple; S + R = spike + ripple + fast ripple; SOZ = seizure onset zone; Res = resection.

The rates of All FR, S + R and S + R + FR were higher inside the resection, compared with outside, for good outcome patients but not for poor outcomes [0.18 (0.07–0.74) versus 0.02 (0–0.31), 0.07 (0.02–0.40) versus 0.05 (0.01–0.08) and 0.51 (0.25–1.61) versus 0.005 (0–0.06), respectively, Fig. 3A-C, *Wilcoxon signed rank P* < 0.05]. The same trend was observed when looking at inside versus outside the SOZ (Table 2). The rates of All S, All R and All FR did not differ (*P* = 0.57, *P* = 0.31 and *P* = 0.23, respectively) between the contacts that were labelled as SOZ and resected and the contacts that were resected but not labelled as SOZ. We also observed that the rate of All S, All R and All FR was double inside the SOZ (and RA) compared with outside areas in good outcome patients (Supplementary Table 4).

**Table 2:** Rates in SOZ/RA versus non-SOZ/non-RA channels by outcome

	Good outcome			Poor outcome		
SOZ [events/min]	Inside	Outside	*P*-values	Inside	Outside	*P*-values
**All S**	1.49 (0.38–8.25)	1.04 (0.20–2.80)	0.002^a^	2.03 (0.68–6.29)	1.39 (0.63–3.33)	0.02^a^
**All R**	1.35 (0.34–3.0)	0.64 (0.31–1.25)	0.002^a^	1.19 (0.32–3.83)	1.0 (0.43–1.81)	0.09
**All FR**	0.35 (0.06–1.35)	0.04 (0.01–0.23)	0.008^a^	0.41 (0.04–2.67)	0.06 (0.02–0.12)	0.055
**S only**	1.45 (0.39–6.88)	1.02 (0.20–2.72)	0.003^a^	1.98 (0.68–3.51)	1.31 (0.58–2.79)	0.07
**R only**	0.70 (0.32–2.01)	0.51 (0.28–0.91)	0.022^a^	0.75 (0.32–1.97)	0.79 (0.36–1.44)	1
**FR only**	0.26 (0–1.39)	0.02 (0.01–0.03)	0.03^a^	0.10 (0.02–0.43)	0.01 (0.002–0.3)	0.016^a^
**S + R**	0.11 (0.001–0.47)	0.05 (0.01–0.07)	0.005^a^	0.18 (0.005–1.17)	0.08 (0.02–0.44)	0.02^a^
**S + FR**	0.01 (0.01–0.51)	0.01 (0–0.09)	0.02^a^	0.03 (0–0.50)	0.01 (0–0.03)	0.08
**R + FR**	0.01 (0–0.17)	0.01 (0–0.31)	0.16	0.06 (0.02–0.17)	0.003 (0–0.02)	0.02^a^
**S + R + FR**	0.07 (0–0.20)	0.01 (0–0.10)	0.03^a^	0.03 (0–0.90)	0.004 (0.001–0.08)	0.08
**S + HFO**	0.11 (0.001–0.66)	0.05 (0.01–0.07)	0.002^a^	0.15 (0.02–1.60)	0.09 (0.02–0.45)	0.009^a^
**RA [events/min]**						
**All S**	1.73 (0.35–8.8)	1.09 (0.16–2.51)	4.1e-5^a^	2.98 (1.16–4.28)	1.28 (0.65–3.40)	0.007^a^
**All R**	1.06 (0.29–2.34)	0.73 (0.32–1.10)	0.02^a^	1.24 (0.52–3.77)	0.86 (0.39–1.88)	0.02^a^
**All FR**	0.18 (0.07–0.74)	0.02 (0–0.31)	0.027^a^	0.43 (0.02–1.69)	0.08 (0.02–0.33)	0.38
**S only**	1.65 (0.37–7.11)	1.07 (0.16–2.48)	3.7e-5^a^	1.91 (1.16–3.68)	1.22 (0.60–2.87)	0.01^a^
**R only**	0.67 (0.25–1.65)	0.57 (0.33–0.99)	0.07	0.82 (0.45–2.39)	0.70 (0.36–1.32)	0.15
**FR only**	0.09 (0–0.12)	0.01 (0–0.04)	0.38	0.08 (0.01–0.36)	0.02 (0.01–0.04)	0.055
**S + R**	0.07 (0.02–0.40)	0.05 (0.01–0.08)	0.003^a^	0.21 (0.05–0.58)	0.06 (0.02–0.45)	0.09
**S + FR**	0.03 (0.02–0.22)	0.01 (0–0.04)	0.06	0.04 (0–0.36)	0.01 (0–0.08)	0.08
**R + FR**	0.02 (0–0.07)	0.018 (0–0.024)	0.58	0.07 (0.01–0.12)	0.007 (0.001–0.02)	0.46
**S + R + FR**	0.51 (0.25–1.61)	0.005 (0–0.06)	0.03^a^	0.03 (0–0.45)	0.004 (0.001–0.21)	0.38
**S + HFO**	0.07 (0.2–0.46)	0.05 (0.01–0.13)	0.003^a^	0.21 (0.09–1.10)	0.06 (0.02–0.46)	0.01^a^

We reported median values for SOZ/RA and non-SOZ/non-RA rates per patient. *P-*value is from the Wilcoxon signed rank test between In-SOZ/In-RA and Out-SOZ/Out-RA groups.

^a^Represented *P-*values <0.05.

The analysis of area percentage revealed that the All S area was comparable in size to the All R area [83.0 (77.6–89.7%) versus 81.5 (67.4–91.4%), *P*≈1, Fig. 3D], but larger than the All FR [7.1 (6.4–39.7%), *P* < 0.01], S + R [18.8 (10.0–26.5%), *P* < 0.01], SOZ [7.5 (3.9–15.5%), *P* < 0.01] and the RA [14.9 (9.0–24.0%), *P* < 0.01]. Similarly, All R area was larger than the All FR (*P* < 0.01), S + R (*P* < 0.01), SOZ (*P* < 0.01) and RA (*P* < 0.01). In contrast, the All FR area was comparable in size to the S + R (*P* = 0.72), SOZ (*P* = 0.99) and RA (*P* = 0.96). The S + R area showed no difference when compared with the SOZ (*P* = 0.14) and RA (*P* = 0.97). Finally, the RA was similar in size to the SOZ area (*P* = 0.54).

### Prediction of SOZ and RA at group level

Supplementary Fig. 2A-B reports the performance of each biomarker in predicting the SOZ and RA for seizure-free patients. At 85% and 90% specificity levels, All FR showed the highest sensitivity (39% and 33%, respectively) and highest global pAUC (0.26) in predicting the SOZ. At 95% specificity, FR only exhibited the highest sensitivity (21%). All the combinations involving spikes and HFOs showed lower sensitivity and pAUC values compared with All S or All FR (Supplementary Fig. 2Ai-iv); All R and R only consistently had the lowest sensitivities (∼17%, ∼12% and ∼9% at 85%, 90% and 95% specificity, respectively) and pAUC (∼0.1).

A similar pattern was observed in predicting the RA (Supplementary Fig. 2B); at specificity levels of 85% and 90%, All FR demonstrated the highest sensitivity (40% and 35%) and pAUC (0.27). At 95% specificity, S + FR exhibited the highest sensitivity (25%). Notably, S + HFO outperformed All S, showing greater sensitivity at 90% and 95% specificity levels (30% and 17%), and a higher global pAUC (0.22).

### Prediction of SOZ and RA at patient level

**Figure 4:**
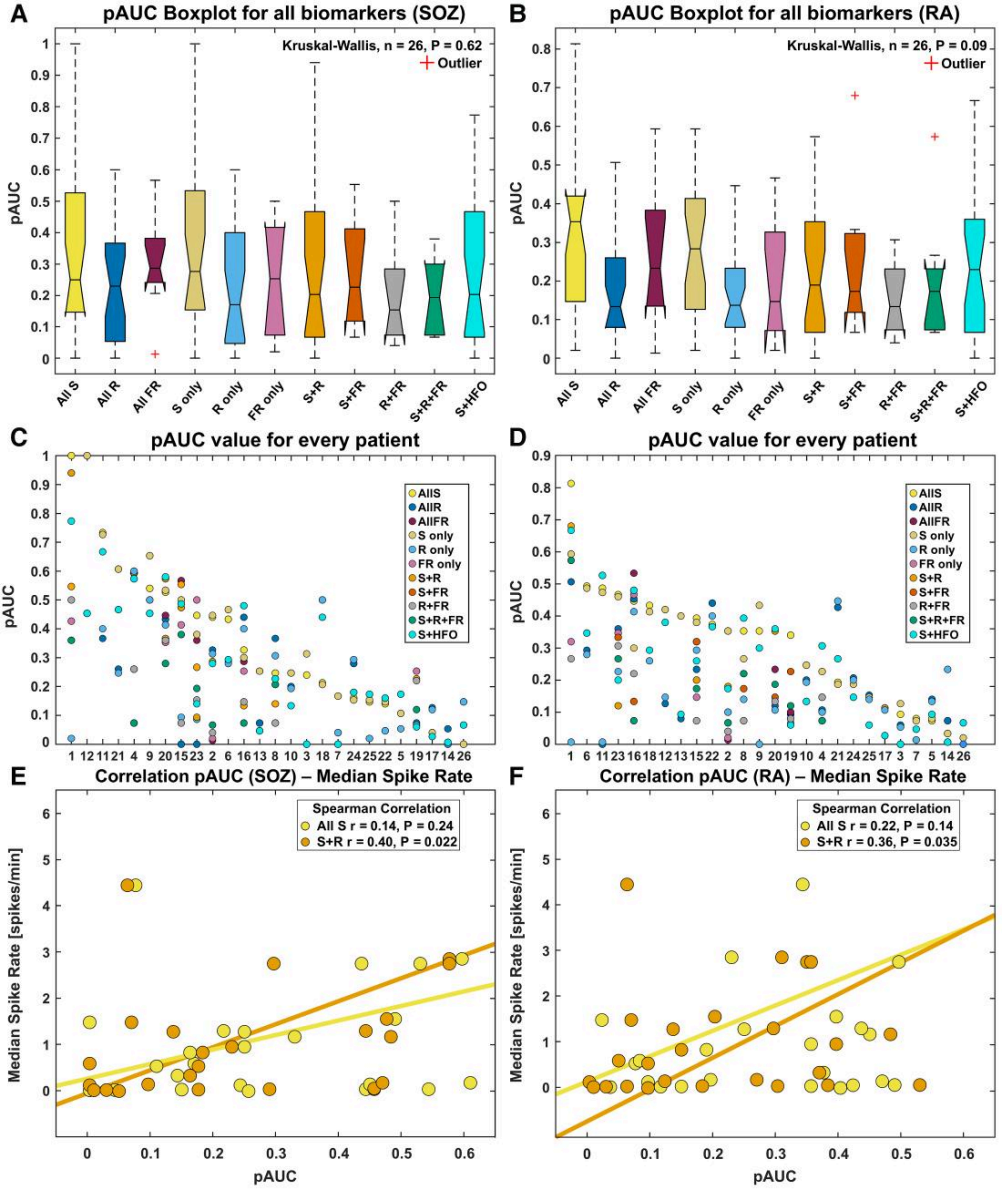
**Partial AUC of each biomarker for prediction of the SOZ (A) and RA (B) at patient level and correlation between pAUC and median spike rate.** (**A,B**) Boxplot of partial AUC (pAUC) for each biomarker. High variance is observed among biomarkers due to patient variability. (**C,D**) Partial AUC for each patient and each biomarker ranked in decreasing order of the All S performance. Noteworthy is the alternating performance of All S and S + R. The dashed horizontal line shows the performance at chance level (0.075). (**E,F**) Spearman Correlation between the pAUC and median spike rate for All S (yellow) and S + R (orange). Note how pAUC for S + R increases with median spike rate for both SOZ and RA prediction. The best fit line was plotted using Matlab function ‘lsline’. All S = all spikes; All R = all ripples, All FR = all fast ripples; AUC = area under the curve; HFO = high-frequency oscillation; pAUC = partial AUC; RA = resected area; R + FR = ripple + fast ripple; S + R = spike + ripple; S + FR = spike + fast ripple; S + R = spike + ripple + fast ripple; S + HFO = spike + HFO; SOZ = seizure onset zone.

Results in this section are reported as median and IQR. In predicting the SOZ, all biomarkers showed similar pAUC values (*Kruskal–Wallis P* = 0.62; Fig. 4A). The same held true when predicting the RA (*Kruskal–Wallis P* = 0.09; Fig. 4B). Figure 4C-D highlights the variable performances for all biomarkers. We repeated this analysis by also including patients who achieved a poor surgical outcome and found similar findings. We observed a positive correlation between the performance of S + R in terms of pAUC and patient’s median spike rate for both SOZ (*r* = 0.40, *P* = 0.022, Fig. 4E) and RA (*r* = 0.36, *P* = 0.035, Fig. 4F) prediction. No correlation was found between the performance of All S in terms of pAUC and patient’s median spike rate for neither the SOZ (*r* = 0.14, *P* = 0.24, Fig. 4E) nor the RA (*r* = 0.22, *P* = 0.14, Fig. 4F) prediction.

### Prediction of surgical outcome

**Figure 5:**
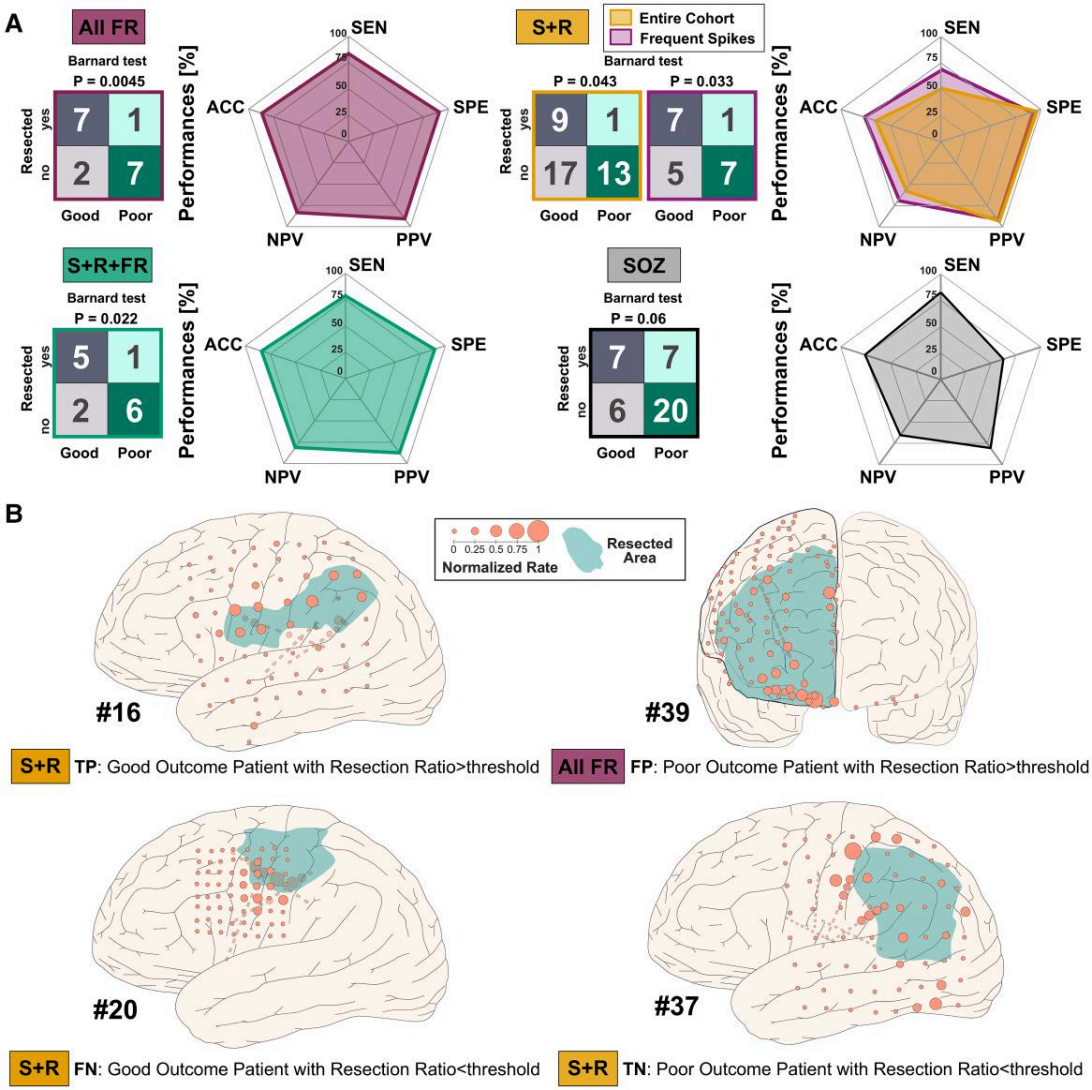
**Outcome prediction using the resection ratio for all fast ripples, spike + ripple, spike + ripple + fast ripple and SOZ ratio.** (**A**) The top panels show the confusion matrices obtained on the entire cohort, selecting the optimal point (maximum Youden Index) on the ROC curve for predicting the post-surgical outcome, as well as sensitivity, specificity, positive predictive value, negative predictive value and accuracy on a radar plot for each biomarker. (**B**) A graphical explanation of the confusion matrix based on the four possible scenarios: (i) the TP is a good outcome 13-year-old boy (#16) with encephalomalacia with the majority of contacts showing S + R that has been resected (i.e. resection ratio >0.5); (ii) the FN is a good outcome 22-year-old female (#20) with centre-parietal focal cortical dysplasia showing only a sub-portion of contacts with S + R that have been resected (i.e. resection ratio <0.5); (iii) the TN is a poor outcome 10-year-old boy (#37) showing only a sub-portion of contacts with S + R that have been resected (i.e. resection ratio <0.5) and, (iv) the FP is a poor outcome 4-year-old boy (#39) with frontal focal cortical dysplasia with the majority of contacts showing All FRs that have been resected (i.e. resection ratio >0.5). On the right side, confusion matrices for All S, All R, All FR and S + R obtained on the whole cohort, setting 0.5 as best threshold for evaluating the post-surgical outcome. SEN = sensitivity; SPE = specificity; PPV = positive predictive value; NPV = negative predictive value; ACC = accuracy.

Table 3 reports the cross-validated ROC curve results for outcome prediction. S + R + FR showed the highest AUC followed by All FR [0.72 (0.69–0.85) versus 0.68 (0.66–0.78), *Wilcoxon rank sum P* < 0.001]. All S and R only showed the highest sensitivity of 92% (*Kruskal–Wallis P* < 0.001). S + R showed the highest specificity followed by All FR [93% (92–100%) versus 88% (86–100%), *Wilcoxon rank sum P* < 0.001]. All FR performed better than other biomarkers in terms of PPV and NPV [M value of 65% (63–78%), *Kruskal–Wallis P* < 0.001]. Moreover, All FR showed the highest accuracy followed by S + R + FR [82% (81–88%) and 79% (77–85%), *Wilcoxon rank sum P* < 0.001]. All FR, S + R and S + R + FR successfully predicted outcome (*Barnard’s test, P* = 0.0045, *P* = 0.04 and *P* = 0.029, respectively, Fig. 5A). No other biomarker was able to predict outcome including the SOZ (*Barnard’s test, P* = 0.06, Fig. 5A). In patients with good outcome, All FR showed higher resection ratio compared with All S (median: 0.69 versus 0.27, *Wilcoxon rank sum P* = 0.018), All R (median: 0.22, *Wilcoxon rank sum P* = 0.006) and S + R (median: 0.32, *Wilcoxon rank sum P* = 0.04). In addition, when focusing on patients with a median spike rate >0.7 spike/min (20, 12 good outcome), S + R predicted outcome (*Barnard’s test*, *P* = 0.033, Fig. 5A) with increased performance; sensitivity increased from 33% to 58%, NPV from 40% to 58% and accuracy from 53% to 70% (Fig. 5A). In patients with no FRs and rare spikes (14; 11 good outcome), we found that: (i) All S correctly predicted outcome in 6 out of 14 patients; (ii) All R predicted outcome in 4 out of 14 patients; and (iii) S + R correctly predicted outcome in 7 out of 14 patients. In our cohort, we explained the confusion matrix for outcome prognostication using three patients with S + R and one with All FRs. Figure 5B shows four examples: (i) a good outcome (TP) and (ii) a poor outcome (TN) patient, predicted correctly based on their S + R resection ratio, a (iii) poor outcome (FP) and a (iv) good outcome (FN) patient, with resection of most contacts recording All FRs and without resection of contacts showing most of S + R, respectively. The pseudo-prospective prediction of outcome, whose results are reported in Supplementary Table 5, showed the best performances for All FR and S + R, with accuracies of 81 (81–81%) and 51 (51–54%), respectively, sensitivities of 78 (75–78%) and 42 (40–44%), respectively and negative predictive values of 78 (75–78%) and 40 (39–42%), respectively.

**Table 3:** ROC analysis for assessment of biomarkers’ performance for prediction of outcome

Biomarker	AUC	SENS [%]	SPEC [%]	PPV [%]	NPV [%]	M	FPR [%]	ACC [%]	J	*P*-values
**All S**	0.57 (0.54–0.61)	92 (35–96)	29 (29–92)	71 (70–90)	67 (39–80)	37 (21–51)	71 (8–71)	70 (51–72)	21 (19–27)	0.067^a^
**All R**	0.53 (0.50–0.57)	73 (72–85)	43 (31–46)	70 (69–73)	46 (42–50)	17 (12–21)	57 (54–69)	63 (62–67)	16 (12–19)	0.17^a^
**All FR**	0.68 (0.66–0.78)	78 (75–88)	88 (86–100)	88 (86–100)	78 (75–88)	65 (63–78)	13 (0–14)	82 (81–88)	65 (63–78)	0.0045^a^
**S only**	0.56 (0.54–0.61)	38 (36–40)	86 (85–92)	83 (41–91)	43 (41–44)	26 (24–34)	14 (7–15)	55 (54–56)	24 (22–31)	0.067^a^
**R only**	0.53 (0.49–0.57)	92 (92–96)	29 (23–31)	71 (70–72)	67 (60–80)	37 (31–51)	71 (69–77)	70 (69–72)	21 (15–25)	0.13^a^
**FR only**	0.63 (0.58–0.73)	71 (67–83)	75 (71–86)	71 (67–83)	75 (71–86)	46 (42–58)	25 (14–29)	73 (71–79)	46 (42–58)	0.062^a^
**S + R**	0.55 (0.52–0.59)	35 (32–36)	93 (92–100)	90 (89–100)	43 (41–45)	33 (31–43)	7 (0–8)	55 (54–56)	27 (25–35)	0.043^a^
**S + FR**	0.59 (0.53–0.69)	75 (71–86)	71 (67–83)	75 (71–86)	71 (67–83)	46 (42–58)	29 (17–33)	73 (71–79)	46(42–58)	0.062^a^
**R + FR**	0.46 (0.40–0.53)	86 (29–100)	25 (25–86)	50 (45–67)	67 (50–100)	17 (0–50)	75 (14–75)	53 (50–57)	11 (0–25)	0.53^a^
**S + R + FR**	0.72 (0.69–0.85)	71 (67–83)	86 (83–100)	83 (80–100)	75 (71–86)	58 (55–75)	14 (0–17)	79 (77–85)	57 (52–71)	0.022^a^
**S + HFO**	0.53 (0.49–0.56)	35 (32–36)	86 (85–92)	82 (80–90)	41 (39–43)	23 (21–31)	14 (8–15)	53 (51–54)	20 (18–27)	0.14^a^
**SOZ**	0.55 (0.55–0.56)	76 (76–77)	50 (50–50)	74 (73–74)	54 (54–54)	27 (27–31)	50 (50–50)	67 (67–69)	26 (26–30)	0.06^a^

^a^Barnard’s Test.

AUC, area under the curve; SENS, sensitivity; SPEC, specificity; PPV, positive predictive value; NPV, negative predictive value; M, markedness; FPR, false positive rate; ACC, accuracy, J, Youden’s index.

## Discussion

Despite spikes being a key biomarker in epilepsy, their role in presurgical evaluation is limited due to their broad presence in non-epileptogenic areas. Here, we show that spikes that temporally overlap with ripples (S + R) can delineate the RA (and the clinically defined SOZ) and predict surgical outcome with high PPV (90%) without having to wait for a seizure to occur. Compared with FR, S + R have lower accuracy, but they are more advantageous since they can be seen in all patients in our cohort. Yet, the accuracy of S + R to delineate the RA and predict outcome is improved, reaching comparable levels with the one of All FR, when patients with frequent spikes are considered. These concepts arise from our primary observations showing that: (i) S + R are observed in all patients while All FR are observed in less than half of our cohort (43%); (ii) good outcome patients show higher rates of All FR, S + R and S + R + FR inside (versus outside) resection, while no differences are seen in poor outcome patients; (iii) All FR, S + R and S + R + FR successfully predict outcome; (iv) S + R presents the highest PPV (90%) followed by All FR (88%); (v) All FR presents the highest accuracy (82%) to predict outcome; (vi) All FR have the highest predictive performance (pAUC and sensitivity) in delineating the SOZ and RA; and (vii) the performance of S + R in terms of pAUC for prediction of SOZ and RA is positively correlated with patients’ median spike rate. Our findings indicate that FRs are the best biomarker, but they can be seen in only half of patients with DRE. S + R are a good alternative with more universal applicability since they can be seen in all patients while their resection predicts good surgical outcome; their performance is improved in patients with frequent spikes.

### Fast ripples are the most accurate biomarker of the RA but is seen in few patients

Fast ripples are more specific biomarker of the RA compared with other interictal biomarkers, such as spikes or ripples.^38,61^ Fast ripples also show higher correlation with the SOZ compared with spikes^9,62,63^ and ripples.^38^ Additionally, surgical removal of cortical tissues that generate fast ripples is linked to better outcome.^33,44^ Yet, fast ripples are observed in only a subset of patients with DRE at rates lower than spikes and ripples. Typically, fast ripple rates are <0.2 events/minute,^38,64^ with only a fraction of the cohort showing their presence.^16,44,65^ For instance, Wang *et al*. reported fast ripple presence in roughly one-third of their cohort,^44^ while van Klink *et al*. reported their presence in more than half patients in their cohort.^65^ Our findings show that only 17 out of 40 patients (43%) had fast ripples, and their occurrence rates were lower than those of spikes (∼1.3 event/min) and ripples (∼0.9 event/min), with an average of 0.1 event/minute. Based on the available literature, the low fast ripple rate in our cohort may be attributed to its heterogeneity; ∼40% of our patients exhibiting fast ripples had FCD, which is typically associated with lower fast ripple rates.^66^ The remaining patients had either normal MRIs or subtle lesions. There is less dense iEEG coverage in these patients (compared with patients with visible MRI abnormalities); this may lead to decreased chances of detecting HFOs due to the limited sampling of several brain regions.^32,67^ Fast ripples are currently considered the leading interictal biomarker for identifying the EZ^38^ with epileptogenic tissue displaying high fast ripple rates, in contrast to non-epileptogenic areas, which typically exhibit very low fast ripple rates.^22^ Our results further corroborate this concept since All FRs performed the best among all other biomarkers in identifying the RA and the SOZ (Supplementary Fig. 2A). At 95% specificity, All FRs and S + FR showed the highest sensitivity and pAUC in the identification of RA contacts (Supplementary Fig. 2B). This is in line with previous studies^14,36^, where All FRs and S + FR had the best sensitivity compared with spikes, ripples and S + R, at 95% of specificity.

### Resection of All FR, S + R and S + R + FR predicts surgical outcome

Resection of brain regions showing All FR, S + R and S + R + FR was linked with good outcome in our cohort. Conversely, the resection ratio of All S and All R did not show an association with good outcome. Our results align with those of numerous independent researchers and prior investigations, in which the removal of fast ripples was consistently shown a statistical association with improved postoperative surgery outcomes across various cohorts and different methods used to study fast ripples.^28,31,65,67^ Fast ripples proved to be the most accurate biomarker for predicting post-surgical outcome, exhibiting an accuracy of more than 80% with 14 out of 17 of correctly identified patients (either good or poor outcome). This is in line with previous studies in the field, which evaluated the accuracy of fast ripples for outcome prediction; these studies reported values ranging from 67% to 81%.^38,68,69^ Outcome prognostication also showed that the resection ratio of S + R predicted good outcome the best, with a PPV of 90% followed by All FR and S + R + FR with a PPV of 88% and 83%, respectively. It is important to note that, outcome prognostication with S + R was performed on the entire cohort, while, for All FR, it was performed on a small subset, therefore reducing its diagnostic and prognostic value. Furthermore, the addition of spikes and ripples to fast ripples does not have additive value, as S + R + FR is seen in even fewer patients, it has lower predictive performance than All FR, and its definition is more time-consuming and resource demanding. Therefore, S + R can be seen as a dependable biomarker, observable in all patients, whose removal is associated with good surgical outcome. Many independent research groups have already highlighted the possibility of considering S + R instead of ripples or spikes occurring in isolation to improve their specificity.^43,44,47,70^ Investigating spikes co-occurring with ripples or vice versa may help to identify only portions of brain seizure-generating tissue.^70^ Our results endorse this notion since S + R is the only biomarker along with All FR and S + R + FR capable of predicting outcome. Moreover, All FR, S + R and S + R + FR were more frequent in the RAs, compared with outside, for good outcome patients only, indicating that the occurrence of these biomarkers delineates pathologic tissue. Note that for poor outcome patients, we observed higher rates of All S and All R inside the RA, while this was not true for the S + R, indicating that a good portion of S + R was not resected. The lower accuracy shown by S + R in outcome prediction compared with All FR can be attributed to the high number of FNs (patients with a S + R resection ratio below threshold that achieved good outcome) observed in this study. Out of the patients classified as FNs, there were only two cases (P2 and P20 in Table 1), where both All FR and S + R failed to predict outcome. In the first case (P2), the epileptogenic focus was in the left mesial frontal region, just anterior to the motor cortex. Resection targeted the left frontal lobe including eloquent cortex related to motor and speech functions. The resection was intentionally limited to avoid potential motor or sensory deficits due to proximity to these areas. Despite incomplete resection, the patient achieved seizure freedom; this may indicate that the critical epileptogenic network was sufficiently disrupted. In the second case (P20, Fig. 5B), the epileptogenic focus was identified in the left sensorimotor region. Corticectomy involved resection of the left-sided precentral gyrus, which corresponded to the cortical dysplasia. The resection was limited to the cortical malformation to minimize functional impairment despite surrounding contacts showing epileptogenic activity. However, some contacts were not resected, as they were located just outside the malformation or situated more superficially. The removal of these two additional contacts would have led to a resection ratio >50%, therefore correctly predicting outcome. This allows us to assume that complete resection of the region showing All FR or S + R is not mandatory to control seizures, indicating that partial resection (or disconnection) is sufficient to impede future generation of seizures. Our pseudo-prospective prediction of outcome further corroborates this notion showing the highest predictive performances for All FR and S + R (Supplementary Table 5).

### S + R has improved the performance of predicting outcome in patients with frequent spikes

We report that S + R identifies the SOZ and RA better than All S. This finding is in line with previous studies where S + R showed a strong association with the clinically defined SOZ,^71,72^ and the best biomarker-value to localize the RA.^43,73^ Yet, at the individual patient level, there was no difference between the pAUC of All S and S + R, consistent with findings from a previous study examining stereotactic EEG in 30 patients with DRE.^14^ Yet, in this previous study, only 10 patients underwent resective surgery; thus, the definition of the EZ for the whole cohort was ambiguous since surgical outcome was not taken into consideration. In our study, correlation analysis showed a positive correlation between the performance of S + R in terms of pAUC for predicting the SOZ or the RA and the median spike rate. This was not true for All S. Therefore, S + R proved to be a better predictor of the SOZ or RA in patients with frequent spikes. These findings are consistent with a previous retrospective study analysing iEEG of 19 patients with DRE,^70^ in which a significant correlation was observed between the median spike rate and performance in terms of pAUC to delineate the SOZ. In this current study, we observed a correlation between median spike rate and pAUC for S + R. Although the correlation was weak (R = 0.4) it was still statistically significant. In contrast, no significant correlation was observed for the ‘all spikes’ category. This suggests that S + R might still serve as a meaningful biomarker, particularly in patients with higher spike rates. Moreover, when we repeated the outcome prediction analysis by including only patients with frequent spikes, the resection ratio of S + R was able to predict outcome with increased performance. More specifically, sensitivity, NPV and accuracy were all increased from 35% to 58%, 44% to 58% and 55% to 70%, respectively. Outcome prediction using the resection ratio of All S remained non-significant in this subset of patients. While performance differed between individuals, S + R were better than All S in patients with frequent spikes. When we repeated the outcome prediction analysis by including patients with no fast ripples and rare spikes, we observed that both All S and S + R performed equally well in predicting outcome. This finding suggests that in such patient subsets, either biomarker can be used effectively to predict outcome with similar accuracy.

### Limitations

All patients included in our cohort had a discrete SOZ, and their seizure localization was clear. Consequently, the study did not explore the connection between spikes, HFOs and the RA in patients with a more intricate epileptic network,^55,74^ such as those with diffuse EEG onset or multifocal epilepsy. Another limitation is the availability of post-surgical outcome at 1 year after surgery and the classification of patients with Engel IB-D score as having good outcome. This limitation is mitigated by the fact that the median follow-up duration of surgical outcome in our cohort was 5 years with outcomes being available for at least 2 years after surgery for 34 (85%) of patients. Notably, 20 patients (77%) who achieved good outcome were completely seizure-free (Engel 1A) at least 1-year after surgery. While most of these patients exhibit long-term seizure freedom indicating that the partial resection/disconnection was effective- we cannot completely rule out the contribution of antiseizure drugs to this outcome particularly in the early postoperative period. Future studies with even longer follow-up periods and more detailed assessments of medication changes are required to further elucidate the relationship between surgical resection, network disconnection and sustained seizure control. Intracranial recordings were analysed at each contact independently from each other; consequently, the propagation pattern of spikes and HFOs was not taken into consideration.^75^ Epilepsy surgery was highly tailored; hence, the surgical outcome was affected by several factors. For this reason, other additive clinical information should also be considered, such as conventional electrophysiological data, imaging findings and pathology. Moreover, the sampling rate for 7 out of 40 patients was ≈ 1000 Hz. While such a sampling rate is not recommended for capturing fast ripples, we assessed the effect of this hardware limitation (Supplementary Table 6), and report that the rate of fast ripples was not affected by this limitation. Finally, the stability of the HFO zone over time could be another possible confounder. HFO rate not only varies between sleep and awake states but also between different sleep stages.^76^ Therefore, it is unknown whether the iEEG duration used in this study is sufficient to capture fully all interictal activity. Prolonging the duration of recording may improve the performance of one biomarker; yet it may negatively affect another one due to signals non-stationarity.

## Conclusions

This study examines the accuracy of various interictal biomarkers in predicting the SOZ or area to resect and, consequently, surgical outcomes. Our results show that S + R are a reliable biomarker of epilepsy since: (i) they are observed in all patients in our cohort; (ii) high S + R are found in the removed tissue of patients who achieved good outcome; (iii) removal of S + R measured by the resection ratio predicts outcome; and (iv) S + R had the highest PPV at 90%. Even though All FR and S + R + FR are more specific to the RA, they cannot be regarded as a singular biomarker of the RA since they are not consistently recorded in all patients with DRE. The combination of spikes and ripples (S + R) seems a promising alternative given that they are both observed in most patients and can automatically be detected with the currently available tools. Yet, this biomarker seems to offer a performance comparable to the fast ripples only in patients with frequent spikes. The combination of different biomarkers is promising but might serve as only an initial step in enhancing the delineation of the RA. Future studies should focus on how to better combine interictal biomarkers by using multivariate classifiers that consider other HFO features or interactions, looking at coupling with slow waves, or including graph theoretical measures.

## Supplementary Material

fcaf056_Supplementary_Data

## Data Availability

The datasets generated during and/or analysed during the current study are available from the corresponding author upon reasonable request. The codes used in this study are available at https://github.com/LorenzoFabbri95/SpikesOnRipples.git.
