# Supplementary material for: Spikes on ripples are better interictal biomarkers of epilepsy than spikes or ripples
Source: Brain Commun. 2025 Feb 8;7(1):fcaf056. doi: 10.1093/braincomms/fcaf056 (PMC11997765; doi:10.1093/braincomms/fcaf056)
Supplement: fcaf056_Supplementary_Data [file fcaf056_Supplementary_Data.docx]

**Supplementary Table 1 | Antiseizure Drugs**

| [#] | **Sex** | **Age** [yr.] | **ASDs** |
| --- | --- | --- | --- |
| **1** | M | 10 | LTG, VPA |
| **2** | M | 17 | LTG, CZP, LZP |
| **3** | M | 10 | OXC, PHT |
| **4** | F | 7 | LCM |
| **5** | F | 13 | LEV, TPM |
| **6** | M | 17 | LZP |
| **7** | F | 16 | LCM, LTG, LEV |
| **8** | M | 2 | VGB, LTG |
| **9** | M | 2.25 | PB, OXC, LEV |
| **10** | M | 19 | LEV, OXC, CLB |
| **11** | F | 18 | LEV, LTG |
| **12** | M | 15 | LEV, LZP, OXC |
| **13** | M | 15 | VPA |
| **14** | F | 18 | ZNS |
| **15** | M | 3 | FBM |
| **16** | M | 13 | LCM, LEV, VPA, LTG, LZP |
| **17** | F | 3 | LTG, CLB, LCM |
| **18** | M | 4 | LEV, CLB, FPHT, VPA, LCM |
| **19** | M | 13 | LCM, OXC, LEV |
| **20** | M | 22 | LCM |
| **21** | F | 18 | LTG, TPM, LEV, LZP |
| **22** | F | 7 | OXC, ZNS, VPA, DZP |
| **23** | F | 12 | OXC, LCM, LEV |
| **24** | M | 15 | LTG, VPA |
| **25** | F | 4 | LTG, OXC, DZP |
| **26** | F | 4 | CLB, LEV, LCM, DZP |
| **27** | F | 9 | FBM, LCM, LEV |
| **28** | F | 9 | RFM, LTG, DZP |
| **29** | M | 6 | VPA, LEV |
| **30** | F | 5 | VPA, FBM |
| **31** | F | 13 | LCM, LEV |
| **32** | M | 17 | OXC, LEV, LTG |
| **33** | F | 15 | DZP, LEV, VPA |
| **34** | M | 12 | LCM, ZNS, OXC, CLB, DZP |
| **35** | M | 16 | CZP, LTG |
| **36** | F | 22 | CLB, LTG |
| **37** | M | 10 | OXC, LEV, LCM, ZNS |
| **38** | F | 6 | ZNS, OXC, DZP |
| **39** | M | 4 | CLB, DZP, LCM |
| **40** | M | 15 | LTG |

LTG = Lamotrigine; VPA = Valproic Acid; CZP = Clonazepam; LZP = Lorazepam; OXC = Oxcarbazepine; PHT = Phenytoin; LCM = Lacosamide; LEV = Levetiracetam; TPM = Topiramate; VGB = Vigabatrin; PB = Phenobarbital; ZNS = Zonisamide; CLB = Clobazam; FPHT = Fosphenytoin; FBM = Felbamate; RFM = Rufinamide; DZP = Diazepam

**Supplementary Table 2 | RippleLab Detection Parameters**

| **Parameter** | **Ripple** | **Fast Ripple** |
| --- | --- | --- |
| Lower Frequency Limit [Hz] | 80 | 250 |
| Upper Frequency Limit [Hz] | 250 | 500 |
| Epoch Length [s] | 60 | 60 |
| SD Threshold | 5 | 5 |
| Min Event Time [ms] | 10 | 10 |

**Supplementary Table 3 | Patients’ Demographics by Outcome**

| **Feature** | **Total** | **Good Outcome Engel I** | **Poor Outcome Engel II-IV** | ***p*** |
| --- | --- | --- | --- | --- |
| Patients, *n* | 40 | 26 | 14 | - |
| Male/female, *n* | 22/18 | 15/11 | 7/7 | 0.74^a^ |
| Age at surgery, years | 12.5 (6-16) | 13 (4.8-16.8) | 11 (6.8-15) | 0.89^b^ |
| Follow-up, years | 5 (2-6) | 4.5 (2-5.8) | 6 (4-6.8) | 0.16^b^ |
| Recording Length^c^ | 5.4 (5-27.7) | 5.9 (5-33.6) | 5.2 (5-5.7) | 0.19^b^ |
| Resection size^d^ | 14.5 (9-26.5) | 14.5 (9.8-26.8) | 18 (8.5-26) | 0.94^b^ |
| Resected Brain Volume^e^ | 2.3 (1.2-3.5) | 2.2 (1.3-3.0) | 2.5 (1.0-5.7) | 0.68^b^ |
| SOZ size^f^ | 9 (4.8-18.5) | 9 (4.3-12.8) | 12 (5.3-21.5) | 0.38^b^ |
| Pathology |  |  |  | 0.27^a^ |
| NL | 9 | 7 | 2 | - |
| DEV | 24 | 13 | 11 | - |
| ACQ | 7 | 6 | 1 | - |

^a^ Bernard test

^b^ Wilcoxon rank sum test;

^c^ Recording Length is reported in minutes

^d^ Resection size is reported as number of removed contacts

^e^ Resected Brain Volume is reported as a percentage of the whole brain

^f^ SOZ size is reported as number of SOZ contacts

M = Male; F = Female; NL = nonlesional; DEV = Malformation of Cortical Development (i.e., focal cortical dysplasia, polymicrogyria, tuberous sclerosis complex, dysembryoplastic neuroepithelial tumor, and glioma); ACQ = Acquired (i.e., stroke, neoplasm, and traumatic brain injury).

**Supplementary Table 4 | Rates in SOZ/RA vs. non-SOZ/non-RA Channels by Outcome**

|  | **Good Outcome** | | | **Poor Outcome** | | |
| --- | --- | --- | --- | --- | --- | --- |
|  | **Inside** | **Outside** | ***p*** | **Inside** | **Outside** | ***p*** |
| 1. **SOZ [events/min]** | | | | | | |
| **All S** | 2.6 (0.73-10.1) | 0.93 (0.23-3.2) | <0.001 | 3.2 (1.1-10.3) | 1.2 (0.39-3.7) | <0.001 |
| **All R** | 1.8 (0.60-4.8) | 0.94 (0.26-2.6) | <0.001 | 2.2 (0.58-8.5) | 0.99 (0.39-2.6) | <0.001 |
| **All FR** | 1.7 (0.4-3.6) | 0.8 (0.2-2.2) | 0.04 | 3 (1.2-5.6) | 1.2 (0.33-4.6) | 0.05 |
| **B. RA [events/min]** | | | | | | |
| **All S** | 2.7 (0.8-10) | 0.8 (0.22-2.8) | <0.001 | 3.3 (1.0-8.5) | 1.1 (0.39-3.2) | <0.001 |
| **All R** | 1.8 (0.35-6.4) | 0.92 (0.28-2.4) | <0.001 | 1.8 (0.65-5.3) | 0.97 (0.38-2.6) | <0.001 |
| **All FR** | 1.2 (0.4-3.6) | 0.6 (0.2-1.9) | 0.002 | 2.0 (0.33-6.0) | 1.8 (0.70-4.6) | 0.64 |

We reported the median value for SOZ/RA and non-SOZ/non-RA rates per patient. *p-*value is from the Wilcoxon signed rank test between In-SOZ/In-RA and Out-SOZ/Out-RA groups. ^*^ represented *p-*values <0.05.

**Supplementary Table 5 | Pseudo Prospective prediction of outcome**

| **Biomarker** | **Correct Predictions** | **SENS [%]** | **SPEC [%]** | **PPV [%]** | **NPV [%]** | **FPR [%]** | **ACC [%]** |
| --- | --- | --- | --- | --- | --- | --- | --- |
| **All S** | 19 (18-19)/39 | 27 (27-28) | 86 (85-86) | 78 (78-78) | 39 (37-40) | 14 (14-15) | 49 (46-49) |
| **All R** | 16 (15-16)/39 | 16 (15-16) | 86 (85-86) | 67 (67-67) | 36 (33-36) | 14 (14-15) | 41 (38-41) |
| **All FR** | 13 (13-13)/16 | 78 (75-78) | 88 (86-88) | 88 (86-88) | 78 (75-78) | 13 (13-14) | 81 (81-81) |
| **S+R** | 20 (20-21)/39 | 42 (40-44) | 71 (71-71) | 73 (71-73) | 40 (39-42) | 29 (29-29) | 51 (51-54) |

We reported the median values and interquartile range from the leave-one-out cross validation.

In this analysis, we investigated how our patients would be classified using a fixed resection ratio threshold of 0.5, based on previous evidence suggesting that complete resection of the entire HFO area may not be necessary for achieving seizure freedom. Specifically, a true positive (TP) was defined as a patient with a good outcome and a resection ratio for a particular biomarker equal to or greater than 0.5, while a true negative (TN) was defined as a patient with a poor outcome and a resection ratio below 0.5. Conversely, a false positive (FP) was classified as a good outcome patient with a resection ratio below 0.5, and a false negative (FN) was a poor outcome patient with a resection ratio equal to or greater than 0.5. A leave-one-out cross-validation was performed by sequentially excluding each patient and repeating the classification process to assess the robustness of our findings. The results, detailed in Supplementary Table 4, indicated that the All FR and S+R approaches demonstrated the highest classification accuracies of 81 (81-81%) and 51 (51-54%), the highest sensitivities of 78 (75-78%) and 42 (40-44%), and the highest negative predictive values of 78 (75-78%) and 40 (39-42%), respectively.

**Supplementary Table 6 | Average frequency of fast ripples sampled at 1000 and 2000 Hz**

|  | **≈ 1000 Hz (n = 4)** | **≈ 2000 Hz (n = 13)** |
| --- | --- | --- |
| **Fast Ripples** | 289 ± 6 Hz | 285 ± 35 Hz |

The peak frequency associated with the ‘island’ or ‘blob’ in the time-frequency plot of each fast ripple was extracted. The fast ripples from the patients sampled at 1000 Hz showed similar mean peak frequency to the fast ripples from the patients sampled at 2000 Hz.

Intracranial EEG recordings were performed with a Natus Quantum amplifier with a high-pass hardware filter having a cutoff frequency of 0.16 Hz, and a low-pass hardware filter having a cutoff frequency of ~340 Hz. The antialiasing frequency of the filters was determined by the Nyquist theorem, corresponding to half of the sampling rate (i.e., 500 Hz for recordings sampled at 1,000 Hz). To evaluate the potential impact of these hardware filters on the results of our study, we analyzed the peak frequency of fast ripples (FRs) to determine whether their frequencies were affected by the filter settings. This analysis was initially conducted on patients sampled at ≥ 2,000 Hz, where FR activity was accurately identified. The average peak frequency of FRs in this cohort, defined as the distinct 'blob' or 'island' in the time-frequency plots, was found to be 285 ± 35 Hz. This peak frequency was similar to that observed in patients sampled at approximately 1,000 Hz (289 ± 6 Hz). These findings suggest that, even at a sampling rate of approximately 1,000 Hz, the low-pass filter with a cutoff near 340 Hz had minimal influence on the detection and characterization of FR activity, supporting the validity of our results across different sampling rates.


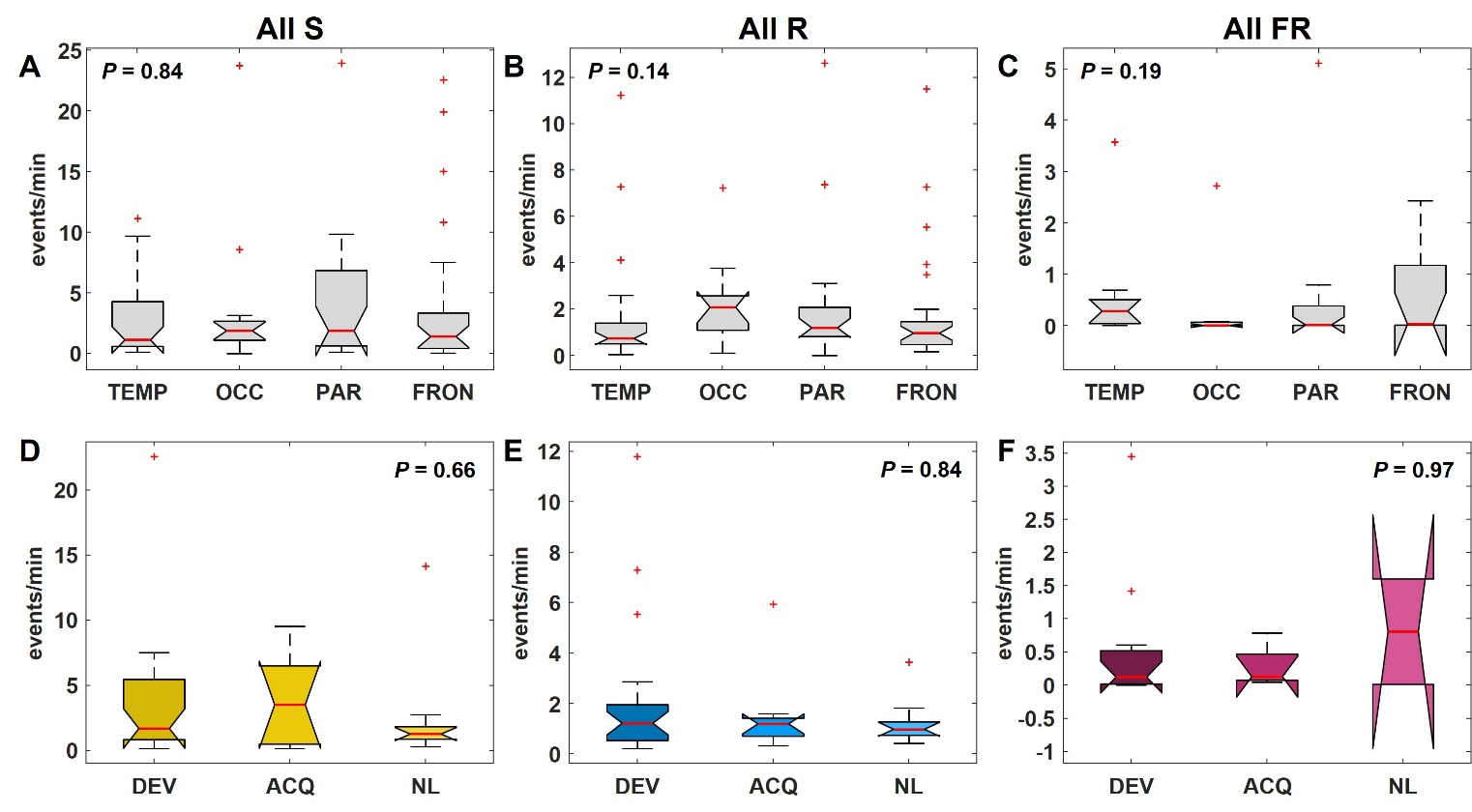


**Supplementary Figure 1.** Comparison of spikes rates (**A**), ripples rates (**B**), and fast ripples rates (**C**) across 4 lobes. Temporal (n=1295), Occipital (n=464), Parietal (n=1158), Frontal (n=1700). Comparison of spikes rates (**D**), ripples rates (**E**), and fast ripples rates (**F**) across 3 groups of underlying conditions. Developmental (n=2647), Acquired (n=931), and Non-Lesional (n=1039). Kruskal-Wallis test was used. Each data point represents, for the top panels, the rate of a channel according to where it was implanted, while for the bottom panels, the rate of a channel according to the patients’ underlying conditions regardless of location. Outliers are plotted as red crosses.


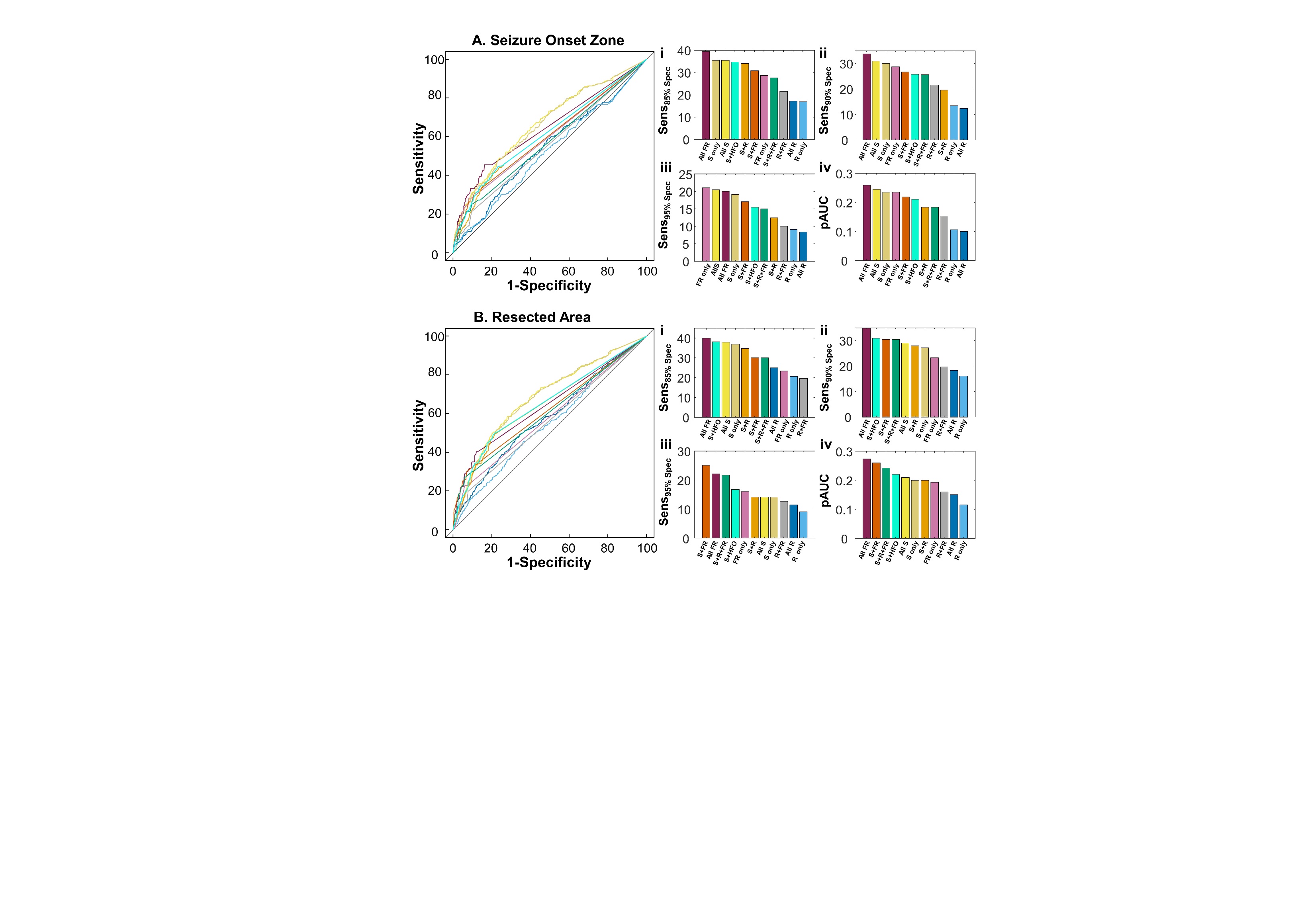


**Supplementary Figure 2. ROC curves for the whole group of good outcome patients for prediction of the SOZ and RA.** The primary **(A-B)** chart displays the ROC curves for each biomarker for prediction of the SOZ and RA respectively. The auxiliary panels **(i-iv)** indicate the sensitivity of individual markers in distinguishing SOZ from non-SOZ channels at specificities of 0.85 (i), 0.90 (ii), and 0.95 (iii), respectively, within the entire cohort of patients with good outcome. The lower panel (iv) represents the pAUC calculated between 0.85 and 1 specificity. All S = All Spikes; All R = All Ripples, All FR = All Fast Ripples; AUC = area under the curve; HFO = High Frequency Oscillation; pAUC = partial AUC; RA = resected area; R+FR = Ripple+Fast Ripple; ROC = receiver operating characteristics; S+R = Spike+Ripple; S+FR = Spike+Fast Ripple; S+R+FR = Spike+Ripple+Fast Ripple; S+HFO = Spike + HFO; Sens = sensitivity; SOZ = seizure onset zone; Spec = specificity.
